# Increasing Azithromycin Resistance in Neisseria gonorrhoeae Due to NG-MAST 12302 Clonal Spread in Canada, 2015 to 2018

**DOI:** 10.1128/aac.01688-21

**Published:** 2022-03-15

**Authors:** Pam Sawatzky, Walter Demczuk, Brigitte Lefebvre, Vanessa Allen, Mathew Diggle, Linda Hoang, Paul Van Caeseele, David Haldane, Jessica Minion, Michael R. Mulvey, Irene Martin

**Affiliations:** a National Microbiology Laboratory, Public Health Agency of Canada, Winnipeg, Manitoba, Canada; b Laboratoire de Santé Publique du Québec, Québec City, Quebec, Canada; c Public Health Ontario Laboratories, Toronto, Ontario, Canada; d Provincial Laboratory for Public Health, Edmonton, Alberta, Canada; e British Columbia Centres for Disease Control Public Health Microbiology & Reference Laboratory, Vancouver, British Columbia, Canada; f Cadham Provincial Laboratory, Winnipeg, Manitoba, Canada; g Queen Elizabeth II Health Sciences Centre, Halifax, Nova Scotia, Canada; h Roy Romanow Provincial Laboratory, Regina, Saskatchewan, Canada

**Keywords:** azithromycin-resistant gonorrhea, NG-MAST, whole-genome sequencing

## Abstract

Azithromycin-resistant (AZIR) gonorrhea has been steadily increasing in Canada over the past decade, which is cause for alarm, as azithromycin (AZI) has been part of the combination therapy recommended by the Canadian Guidelines on Sexually Transmitted Infections (CGSTI) since 2012. Neisseria gonorrhoeae with AZI MICs ≥1 mg/L collected between 2015 and 2018 as part of the Gonococcal Antimicrobial Surveillance Program-Canada underwent antimicrobial susceptibility testing, molecular typing, and whole-genome sequencing. Regional, demographic, and clinical isolation site comparisons were made to aid in our understanding of AZI susceptibility trending. We identified 3,447 N. gonorrhoeae with AZI MICs of ≥1 mg/L in Canada, which increased from 6.3% in 2015 to 26.5% of isolates in 2018. Central Canada had the highest proportion, rising from 9.2% in 2015 to 31.2% in 2018. We identified 273 different N. gonorrhoeae multiantigen sequence types (NG-MAST) among these isolates, with ST-12302 the most prevalent (50.9%). Whole-genome sequencing identified the Neisseria lactamica-like mosaic *mtr* locus as the mechanism of AZIR in isolates of ST-12302 and isolates genetically similar (differing by ≤5 bp), designated the ST-12302 genogroup, accounting for 65.2% of study isolates which were originally identified in central Canada but spread to other regions by 2018. Genomic analysis indicated that AZIR in Canadian N. gonorrhoeae expanded rapidly due to clonal spread of the ST-12302 genogroup. The rapid expansion of this AZIR clonal group in all regions of Canada is of concern. CGSTI are currently under review to address the increase in AZIR in Canada.

## INTRODUCTION

Gonorrhea, caused by Neisseria gonorrhoeae, is the second most commonly reported bacterial sexually transmitted infection in Canada, increasing from 21.8 cases per 100,000 population in 2001 to 94.3 cases per 100,000 in 2018 (1). An estimated 87 million cases of gonorrhea are identified globally each year (2). N. gonorrhoeae easily acquires genetic mutations, resulting in the rapid development of resistance to antibiotics, which complicates treatment and control. In response to the increase in antimicrobial resistance (AMR) in N. gonorrhoeae, the World Health Organization (WHO) published a global action plan to attempt to contain its spread (3), and the CDC labeled drug-resistant N. gonorrhoeae as an urgent hazard (4). Reported treatment failures and isolates with decreased susceptibility to extended-spectrum cephalosporins (ESC) in Canada (5) prompted the 2012 update of Canadian Guidelines on Sexually Transmitted Infections to recommend combination therapy with 2 antibiotics, ceftriaxone or cefixime and azithromycin (AZI) as first-line treatment for high-risk cases (https://www.canada.ca/en/public-health/services/infectious-diseases/sexual-health-sexually-transmitted-infections/canadian-guidelines.html).

The Canadian Gonococcal Antimicrobial Surveillance Program (GASP-Canada), initiated in the mid-1980s, is a culture-based laboratory surveillance system monitoring AMR trends and N. gonorrhoeae multiantigen sequence types (NG-MAST). Over the last few years and since the introduction of combination therapy for the treatment of gonorrhea in Canada, laboratory surveillance has reported that decreased susceptibility to the ESCs has been declining in Canada, but AZI resistance (AZIR; MIC ≥ 2 mg/L) has been increasing, with 0.9% (26/3036) in 2012 to 7.6% (427/5,607) in 2018 (1). Increases in AZIR have been documented in other countries as well, including the United States (6), United Kingdom (7–9), Australia (10), and Japan (11). Treatment failures have also been documented in Canada (12), the United Kingdom (13, 14), and Australia (14). AZIR rates have now exceeded WHO thresholds of 5% in some regions of the world, warranting changes to the prescribed gonorrhea treatments (3).

There is some variability in the AZIR breakpoints being used globally. In 2019, the Clinical and Laboratory Standards Institute (CLSI) defined AZI susceptibility for N. gonorrhoeae as ≤1 mg/L but did not establish an MIC indicating resistance (15). The European Committee on Antimicrobial Susceptibility Testing (EUCAST [https://www.eucast.org/clinical_breakpoints]) reported the epidemiological cutoff value for azithromycin to be 1 mg/L (16). Although GASP-Canada uses an MIC of ≥2 mg/L as the AZIR breakpoint, as agar dilution MICs are accepted within ±1-log dilution, in this study, we included all isolates with MICs of ≥1 mg/L, and for convenience, these isolates are referred to as AZIR.

Gene mutations associated with AZIR have been widely studied (17–25). Resistance mechanisms identified include point mutations in 23S rRNA; overexpression of the MtrCDE efflux pump due to various mutations; the less common presence of *erm* genes, which encode 23S rRNA methylases; inactivation of macrolides by esterases or phosphotranserases (encoded by *ere* and *mph* genes, respectively); the *mef* and MacAB efflux pumps; and ribosomal gene mutations in *rplD* and *rplV*.

More recently, mosaic multiple transferable resistance (*mtr*) efflux pump alleles, originating through horizontal gene exchange from N. lactamica and N. meningitidis, have been linked to increased resistance to AZI (25). AZIR gonorrhea with mosaic *mtr* alleles have been documented in Canada (20), the United States (26, 27), Australia (28), and Germany (29).

Through GASP-Canada, we collected AZIR N. gonorrhoeae isolated in Canada between 2015 and 2018. Phenotypic, demographic information, and whole-genome sequencing (WGS) data were analyzed to aid in our understanding of AZIR trends and the molecular epidemiology of these isolates.

## RESULTS

**FIG 1:**
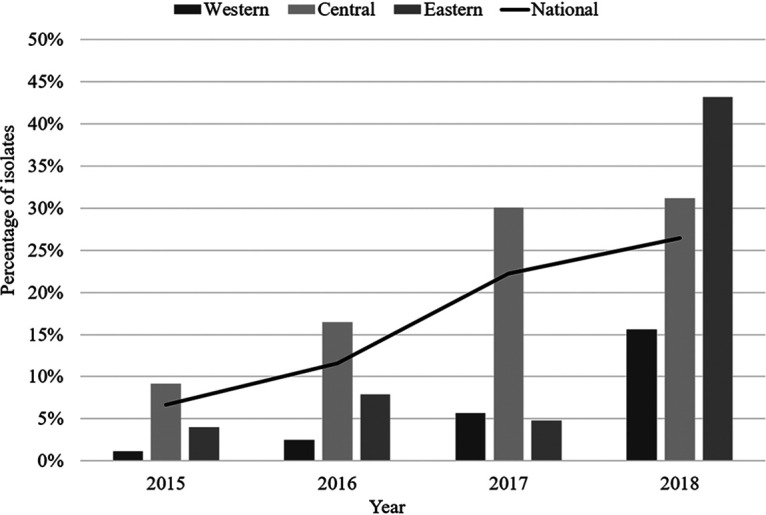
Annual distribution of N. gonorrhoeae isolates with azithromycin MICs of ≥1 mg/L by region, 2015 to 2018. Denominators used to determine percentages are the number of cultures tested in each province as follows: for 2015, western, 1,506; central, 2,659; and eastern, 25; for 2016, western, 1,568; central, 2,932; and eastern, 38; for 2017, western, 1,654; central, 3,594; and eastern, 42; and for 2018, western, 1,708; central, 3,955; and eastern, 44. National percentages based on total number of isolates tested nationally as follows: 4,190 isolates in 2015, 4,538 in 2016, 5,290 in 2017, and 5,607 in 2018.

From 2015 to 2018, the number of N. gonorrhoeae isolates identified with azithromycin MICs of ≥1 mg/L (AZIR) increased each year from 6.3% in 2015 (262/4,190) to 26.5% in 2018 (1,486/5,607, *P* < 0.05) (Table 1; Fig. 1). The central region of Canada had the highest number of these isolates, from 9.2% (244/2,659) in 2015, rising to 31.2% (1,201/3,855, *P* < 0.05) in 2018. In the western region, 1.1% (17/1,505) of isolates were AZIR in 2015, which increased to 15.6% (266/1,703, *P* < 0.05) in 2018. Eastern provinces saw an increase from 4.0% (1/25) in 2015 to 43.2% (19/44, *P* < 0.05) in 2018.

**TABLE 1:** N. gonorrhoeae isolates tested in Canada, 2015 to 2018

Yr	No. of isolates tested nationally	No. of isolates with AST data	Isolates with AZ MIC of ≥1 mg/L		
			Total no. of isolates with AZ MIC of ≥1 mg/L	No. (%) of isolates with NG-MAST data^*a*^	No. (%) of isolates with WGS data
2015	4,190	2,638	262	262 (100)	56 (21.4)
2016	4,538	3,092	524	512 (97.7)	105 (20.0)
2017	5,290	4,143	1,175	1,137 (96.8)	682 (58.0)
2018	5,607	4,943	1,486	1,466 (98.7)	701 (47.2)
Total	19,625	14,816	3,447	3, 377 (98.0)	1,544 (44.8)

a Some isolates were nontypeable or not done (submitted data); denominator used is total number of isolates with AZ MIC of ≥1 mg/L.

### Antimicrobial resistance.

The AZI MIC for over 50% of study isolates was 1 mg/L (54.45%, 1,877/3,447) and ranged to ≥512 mg/L (0.09%, 3/3,447); 37.10% (1,279/3,447) had an MIC of 2 mg/L. In addition to having low-level AZIR, 95.79% (3,302/3,447) were resistant to at least 2 other antibiotics, including erythromycin, ciprofloxacin, and tetracycline, identifying them as multidrug resistant (MDR).

### NG-MAST.

**FIG 2:**
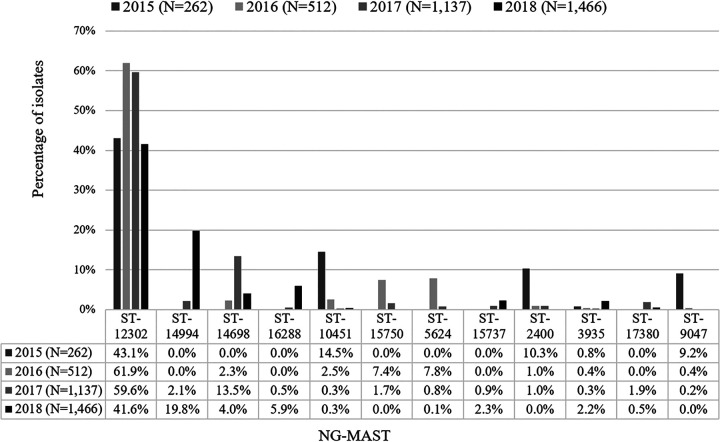
Prevalent NG-MAST of Canadian N. gonorrhoeae isolates with azithromycin MICs of ≥1 mg/L by year, 2015 to 2018 (*n* = 3,377), with 2,700 isolates represented. The remaining 677 isolates were dispersed among 261 different STs.

There were 273 different NG-MAST sequence types identified among the 3,377 AZIR isolates typed. The most prevalent ST was ST-12302 overall (50.9%, 1,718/3,377), and in all 4 years, its prevalence was 43.1% in 2015 (113/262), 61.9% in 2016 (317/512), 59.6% in 2017 (678/1,137), and 41.6% in 2018 (610/1,466). ST-12302 is responsible for the overall increase in AZIR in Canada. ST-14994 is the next most prevalent ST (9.3%, 315/3,377), only identified among study isolates in 2017 (2.1%, 24/1,137) and 2018 (19.8%, 291/1,466). ST-14698 (6.6%, 223/3,377) followed with 2.3% (12/512) in 2016, 13.5% (153/1,137) in 2017, and 4.0% (59/1,466) in 2018 (Fig. 2). ST-14698 differs from ST-12302 by less than 5 bp and is therefore part of the ST-12302 genogroup (ST-12302g). There was a total of 2,250 ST-12302g isolates (66.6%, 2,250/3,377), accounting for 43.9% in 2015 (115/262), 74.6% in 2016 (382/512), 83.3% in 2017 (947/1,137), and 55.0% in 2018 (806/1,466) of study isolates (Table 2). ST-16288 (2.8%, 93/3,377), first identified in 2017 (0.5%, 6/1,137) and 2018 (5.9%, 87/1,466), was the fourth most prevalent ST identified among AZIR isolates (Fig. 2). Isolates of all four of the above prevalent STs predominantly had low-level AZIR with MICs of 1 to 2 mg/L.

**TABLE 2:** Prevalent NG-MAST sequence types of Canadian N. gonorrhoeae isolates with azithromycin MICs of ≥1 mg/L by year and region, 2015 to 2018 (*n* = 3,377)

NG-MAST sequence type	No. (%) of isolate in:											
	2015 (*n* = 262)			2016 (*n* = 512)			2017 (*n* = 1,137)			2018 (*n* = 1,466)		
	Western (*n* = 17)	Central (*n* = 244)	Eastern (*n* = 1)	Western (*n* = 26)	Central (*n* = 483)	Eastern (*n* = 3)	Western (*n* = 57)	Central (*n* = 1,078)	Eastern (*n* = 2)	Western (*n* = 257)	Central (*n* = 1,190)	Eastern (*n* = 19)
ST12302g	0 (0)	115 (43.9)	0 (0)	9 (1.8)	371 (72.5)	2 (0.4)	30 (2.6)	915 (80.5)	2 (0.2)	92 (6.3)	699 (47.7)	15 (1.0)
ST14994	0 (0)	0 (0)	0 (0)	0 (0)	0 (0)	0 (0)	0 (0)	24 (2.1)	0 (0)	17 (1.2)	271 (18.5)	3 (0.2)
ST16288	0 (0)	0 (0)	0 (0)	0 (0)	0 (0)	0 (0)	3 (0.3)	3 (0.3)	0 (0)	83 (5.7)	4 (0.3)	0 (0)
Other	17 (6.5)	129 (49.2)	1 (0.4)	17 (3.3)	112 (21.9)	1 (0.2)	24 (2.1)	136 (12.0)	0 (0)	65 (4.4)	216 (14.7)	1 (0.1)

Regionally, ST-12302g (including ST-12302, ST-14698, ST-15750, ST-17380 plus 57 other STs) was most prevalent in central Canada in all 4 years (in 2015, 43.9% [115/262]; in 2016, 72.5% [371/512]; in 2017, 80.5% [915/1,137]; and in 2018, 47.7% [699/1,466]). The eastern and western provinces identified ST-12302g isolates in 2016 (0.4% [2/512] and 1.8% [9/512], respectively), 2017 (0.2% [2/1,137] and 2.6% [30/1,137], respectively), and 2018 (1.0% [15/1,466] and 6.3% [92/1,466], respectively) (Table 2).

AZIR ST-14994 isolates were identified in central Canada in 2017 (2.1%, 24/1,137) and 2018 (18.5%, 271/1,466) and in western (1.2%, 17/1,466) and eastern provinces (0.2%, 3/1,466) in 2018. ST-16288, first identified in 2017 in western (0.3%, 3/1,137) and central (0.3%, 3/1,137) provinces, was the third most prevalent ST in 2018 (5.9%, 87/1,466), with four isolates in central Canada (0.3%, 4/1,466) and 83 in western Canada (5.7%, 83/1,466) (Table 2).

### Whole-genome sequencing analysis.

The Illumina MiSeq platform (Illumina, San Diego, CA) generated paired-end, 300-bp indexed reads with an average genome coverage of 86 times and an average of 1,104,350 reads per genome. The average contig length generated was 23,043 bp, and the average *N*_50_ contig length was 62,832 bp.

A summary of mutations associated with AZIR can be found in Table S1 in the supplemental material. The prevalent mutations identified include mosaic *mtrR* promoter (as described by Rouquette-Loughlin [24]) (95.0%, 1,467/1,544), *mtrR* mutations D79, S183N, and M197I (92.2%, 1,423/1,544), mosaic *mtrC* (95.1%, 1,468/1,544), *mtrD* mutations S821A and K823E (95.2%, 1,470/1,544), mosaic *mtrE* (92.8%, 1,433/1,544), *porB1b* G120K mutation (73.8%, 1,139/1,544), *rpsJ* V57M (99.7%, 1,539/1,544), and *rplD* V125A, A147G, and R157Q (92.9%, 1435/1544). Two isolates had the *mtrD* K823E mutation but not the S821A.

The 23S rRNA C2611T mutation was present in 6.2% (96/1,544) of study isolates with WGS results as follows: 32.1% (18/56) in 2015, 11.4% (12/105) in 2016, 3.5% (24/682) in 2017, and 6.0% (42/701) in 2018. AZI MICs of these isolates ranged from 1 mg/L to ≥512 mg/L, although only 2 isolates were ≥512 mg/L. These 2 isolates had C2611T on four alleles and had the mosaic *mtr* locus as well. Seven other isolates with the same mutations had AZI MICs of 32 or 64 mg/L. Of isolates with the C2611T mutation, 56.3% (54/96) had mutations on all 4 alleles, 21.9% (21/96) on 3 alleles, 15.6% (15/96) on 2 alleles, and 4.2% (4/96) on only 1 allele. The 23S rRNA A2059G mutation was identified in only 3 isolates, and each of those had the mutation on all 4 alleles. They were isolated in 2017 (*n* = 1) and 2018 (*n* = 2) and had AZI MICs of ≥512 mg/L. The 23S rRNA A2058G mutation was not identified in our study isolates.

### WGS analysis of ST-12302g.

WGS was performed on 1,259 (56.0%) ST-12302g isolates in this study with the most prevalent multilocus sequence type (MLST) and N. gonorrhoeae sequence typing for antimicrobial resistance (NG-STAR) types being 9363 (94.0%, 1,183/1,259) and 168 (80.0%, 1,009/1,259), respectively. Mosaic forms of the *mtrR* promoter (as described by Rouquette-Loughlin et al. [24]) plus mosaic *mtrR* with the D79N, S183N, and M197I mutations and *mtrD* with the S821A and K823E mutations (24) were identified. Mosaic *mtrC* and *mtrE* alleles with 3.88% (16/412) and 3.21% (15/467) of the gene having amino acid (aa) mutations compared to the reference strain WHO F, respectively, were also identified. *mtrF* alleles were nonmosaic. All isolates had the PorB1b protein structure, with 84.8% (1,068/1,259) having the G120K mutation; the remaining 15.2% (191/1,259) had the G120N mutation (Table 3). No 23S rRNA A2058G or A2059G mutations were identified in this group, but 2.5% (32/1,259) had 23S rRNA C2611T mutations in 1 to 4 alleles. No mutations were identified in the *ponA*, *rplV*, or *macA* genes. The *rplD* G70D mutation was not identified in any of these isolates, and none of the isolates had *ermA*, *ermB*, or *ermC*; however, two isolates (0.2%, 2/1,259) had *ermF*. No Correia element or *ereA*, *ereB*, *mefA*, or *mphA* genes were identified. Analysis by Pathogenwatch (pathogen.watch) confirmed the presence or absence of these mutations and genes and classified the mosaic *mtrR* promoter and *mtrD* mutations as N. lactamica-like or type 2 (*mtrD* mosaic 2; *mtrR* promoter mosaic 2) (30).

**TABLE 3:** Mutations, other than 23S rRNA mutations, affecting azithromycin MICs of prevalent NG-MAST sequence types in this study, 2015 to 2018 (*n* = 3,377)

NG-MAST sequence type	Yr (no. of isolates)					*mtrR* mutations^*a*^					*mtrD* mutations		*mtrC*	*mtrE*	Other mutations related to AZIR			Avg AZ MIC^*d*^
	2015	2016	2017	2018	Total	A39T	Mosaic promoter^*b*^	Mosaic			Mosaic		Mosaic	Mosaic				
								D79N	S183N	M197I	S821A	K823E	% aa differences^*c*^	% aa differences^*c*^	*porB1* structure	*porB1* G120	*ponA*	
ST12302g^*e*^	115	382	947	806	2,250		X^*g*^	X	X	X	X	X	3.88 (16/412)	3.21 (15/467)	IB	K^*f*^		1.41 (*n* = 790)
ST14994	0	0	24	291	315	X	X				X	X	3.64 (15/412)	2.14 (10/467)	IA		L421P	1.08 (*n* = 290)
ST16288	0	0	6	87	93		X	X	X	X	X	X	3.88 (16/412)	3.21 (15/467)	IB			1.06 (*n* = 87)

a No G45D mutations were identified in prevalent study isolates; there were 8/1,544 identified in nonprevalent NG-MAST types.

b Mosaic promoter mutations as described by Rouquette-Loughlin et al. (24).

c Percent represents the number of amino acid (aa) mutations/size of gene.

d Only MICs from 1 to 4 mg/L of 2018 isolates used to determine mean to exclude isolates with 23S rRNA mutations, which generally have higher MICs.

e Fifty-nine STs with less than 5 base pair differences than ST-12302; mutations indicated are for majority of isolates in the genogroup.

f G120K ≈ 85%, G120N ≈ 15%.

g “X” indicates the presence of the mutation.

### WGS analysis of ST-14994.

The 42 ST-14994 isolates with WGS were MLST 7822 (100%, 42/42) and NG-STAR 1493 (97.62%, 41/42), and all had the mosaic *mtrR* promoter identical to that of ST-12302g and mosaic *mtrD* alleles with the S821A and K823E mutations (classified by Pathogenwatch as N. lactamica-like or type 2). Their *mtrC* allele was mosaic but with 1 less mutation (3.64%, 15/412) than the ST12302g *mtrC* allele; the *mtrE* allele was partially mosaic, with 2.14% (10/467) amino acid differences from WHO F compared to the ST12302g *mtrE* allele (3.21%, 15/467). Unlike ST-12302g, they did not have mosaic *mtrR* alleles but did have the *mtrR* A39T mutation, the *ponA* L421P mutation, and the PorB1a structure and, therefore, no *porB* mutations (Table 3). No other AZIR-related mutations or genes were identified except for one isolate with the 23S rRNA C2611T mutation on all 4 alleles and an AZI MIC of 32 mg/L.

### WGS analysis of ST-16288.

ST-16288 (88.2%, 82/93 with WGS data) differs from ST-12302 by 34 bp but has the same *tbpB* (*tbpB*-267). Isolates are MLST 9363 and NG-STAR 1487 and have the same mutations as ST-12302 isolates except they have no *porB*1b mutations (Table 3).

### Phylogenetic analysis.

**FIG 3:**
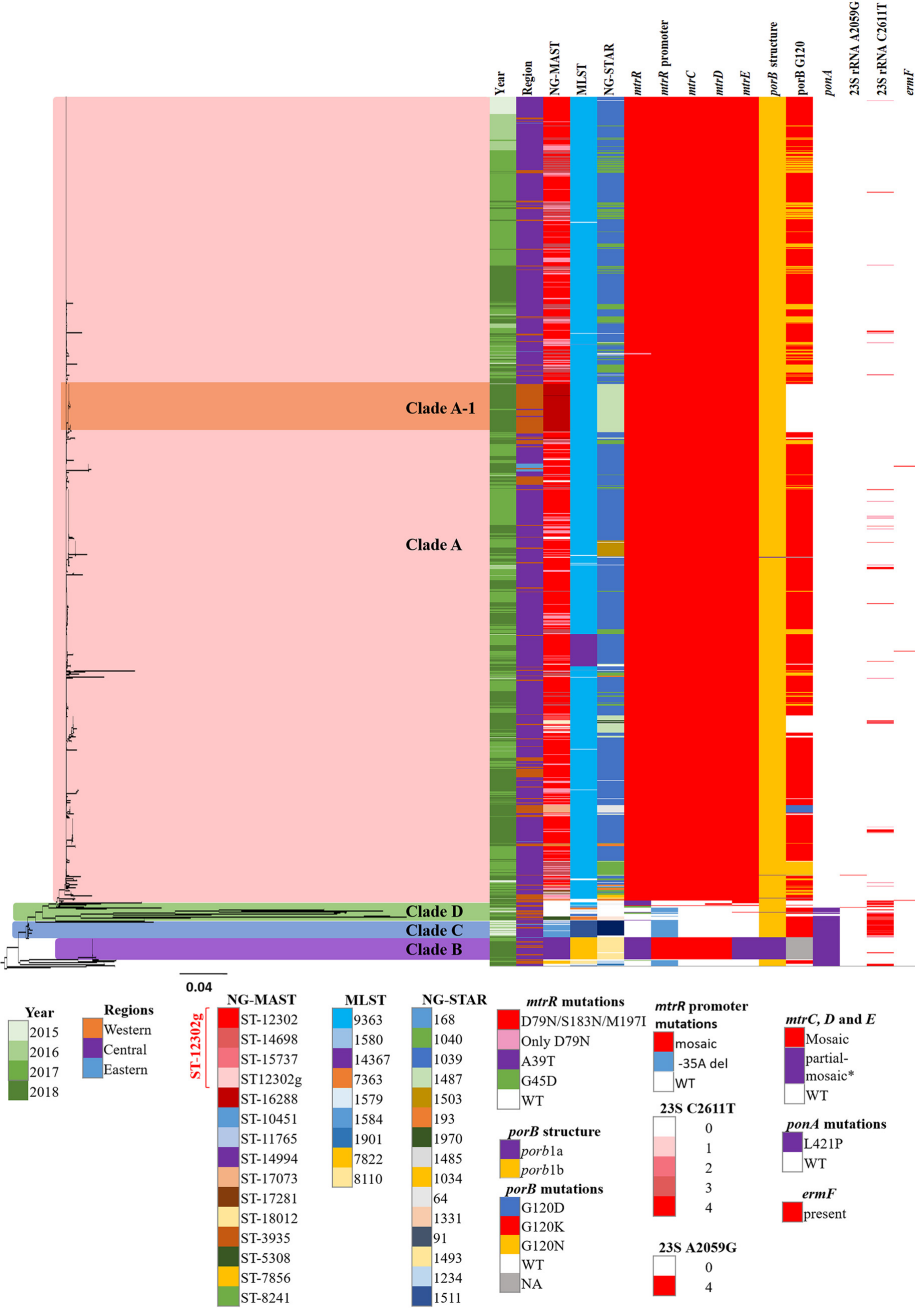
Whole-genome core SNP maximum-likelihood phylogenetic tree of 1,477 N. gonorrhoeae isolates with AZI MICs of ≥1 mg/L collected from 2015 to 2018 in Canada, including NCCP11945 as the reference strain. The scale bar represents the estimated evolutionary divergence between isolates on the basis of the average pairwise distance between strains. Year, region, STs, and mutations related to AZIR are indicated. Clade A is primarily NG-MAST 12302g, excluding clade A-1c, which is NG-MAST 16288 and differs from ST-12302 by 39 bp. Clade B is represented by NG-MAST 14994, Clade C is primarily NG-MAST 10451, and Clade D is fairly diverse.

**FIG 4:**
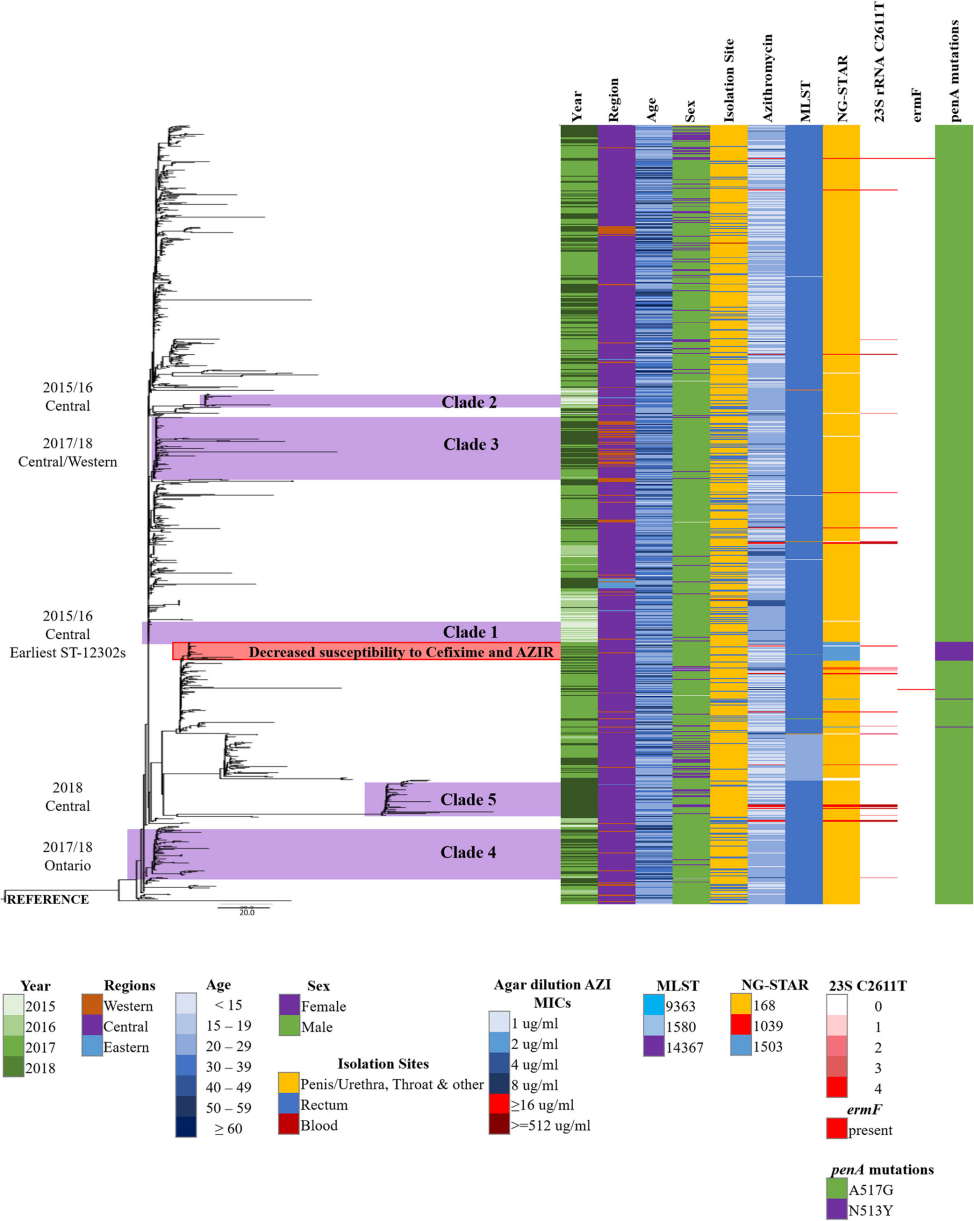
Whole-genome core SNP maximum-likelihood phylogenetic tree of 911 Neisseria gonorrhoeae isolates with AZI MICs of ≥1 mg/L and NG-MAST 12302 collected from 2015 to 2018 in Canada. The scale bar represents the estimated evolutionary divergence between isolates on the basis of the average pairwise distance between strains. Year, region, age, sex, isolation sites, AZI MICs, MLSTs, NG-STARs, and 23S rRNA C2611T, *ermF*, and *penA* mutations are indicated. Clades 1 and 2 contain the oldest ST-12302 isolates and are ancestral to the strains in the ST-12302 genogroup.

Phylogenetic analysis (Fig. 3) of 1,477 of the study isolates exhibited 4 major clades, with the largest clade (A) representing 92.6% (1,368/1,477) of the isolates in the tree. Isolates located in clade A are primarily of the NG-MAST ST-12302g and have the associated molecular types, AMR, and mutations. Phylogenetic analysis of 911 ST-12302 isolates (Fig. 4) provided further resolution of strains and differentiated the isolates temporally, geographically, and by risk group.

The earliest ST-12302 isolate was identified in central Canada in May of 2015. It was an ST not previously identified, comprised of *por*-908 and *tbpB*-267. In Fig. 4, clade 1 (*n* = 32) largely contained the earliest isolates, 59.4% (19/32) from 2015 and 40.6% (13/32) from 2016. One clade 1 isolate was from western Canada, with the remaining from central Canada (96.9%, 31/32); 93.8% (30/32) of clade 1 isolates are from males with 30.0% (9/30) rectal isolation sites. Clade 2 isolates (*n* = 14), collected in 2015 (50.0%, 7/14) and 2016 (50.0%, 7/14), were also primarily from central Canada (85.7%, 12/14), all from males, and 28.6% (4/14) are rectal isolates. These 2 early clades, associated geographically in central Canada, differ by an average of 22 single nucleotide variants (SNVs) (range of 21 to 26 SNVs) and were ancestral to the other isolates circulating in Canada.

Clade 3 isolates were from central Canada (58.7%, 37/63) and the western provinces (41.3%, 26/63). This particular cluster started in central Canada in late 2016 and spread to western regions in late 2017 continuing into 2018. The isolates were primarily from males (96.8%, 61/63), with rectal infections responsible for over one-third of male isolates (36.1%, 22/61).

Clade 4 isolates were identified in both 2017 (53.4%, 31/58) and 2018 (46.6%, 27/58), primarily in central Canada (94.8%, 55/58), with 5.2% (3/58) from western Canada, mostly from males (96.6%, 56/58), with 21.4% (12/56) rectal isolates. Clade 5 isolates were identified solely in 2018 and primarily in central Canada (97.6%, 40/41). One isolate was identified in eastern Canada. The proportion of males was lower in this clade (75.6%, 31/41) and with only 9.7% from rectal infections. Other clades not noted show the increase in isolates from females in 2017 and 2018.

**FIG 5:**
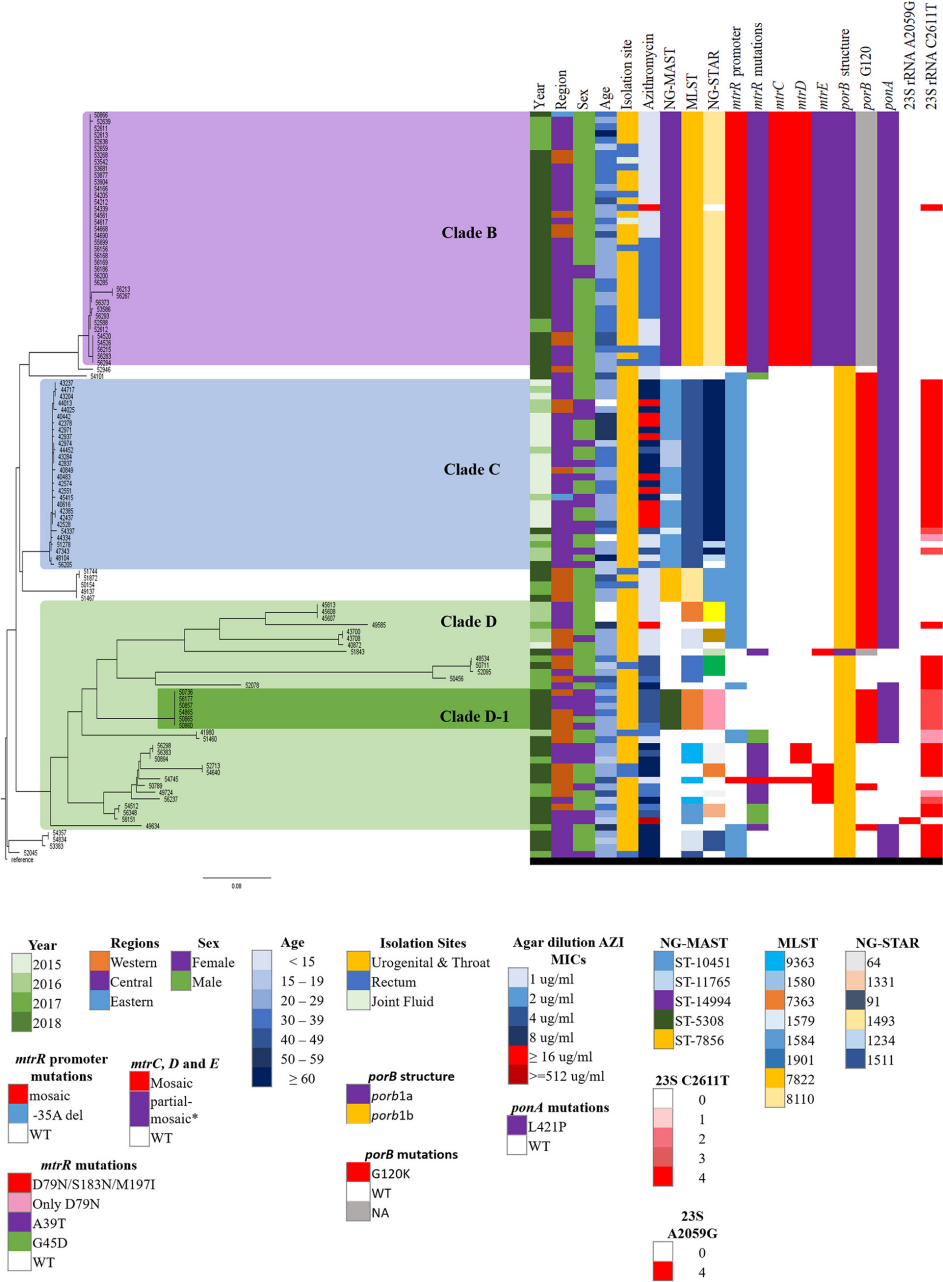
Whole-genome core SNP maximum-likelihood phylogenetic tree of 112 N. gonorrhoeae isolates with AZI MICs of ≥1 mg/L that are not part of clade A of Fig. 1, collected from 2015 to 2018 in Canada, including the oldest outlier (NML number 51307) as the reference strain. The scale bar represents the estimated evolutionary divergence between isolates on the basis of the average pairwise distance between strains. Year, region, sex, age, isolation site, AMR, AZI MICs, STs, and mutations related to AZIR are indicated. Clade B is represented by NG-MAST 14994, Clade C by NG-MAST 10451g, and Clade D is fairly diverse. Clade D-1 is represented by ST-5308 and is AZIR and has decreased susceptibility to extended-spectrum cephalosporins (ESCDS).

Figure 5 further differentiates isolates that clustered in the bottom section of the tree in Fig. 3. Clade B are all NG-MAST-14994 (*n* = 38), first identified in 2017 (18.2%, 7/38), and they were primarily from males (94.7%, 36/38), with 22.2% (8/36) of these from rectal infections.

All isolates in clade C were either ST-10451 or within 5 bp of ST-10451 (*n* = 28) and were identified primarily in 2015 (60.7%, 17/28) in central Canada (85.7%. 24/28). A total of 57.1% (16/28) were from males and none from rectal infections. These isolates have the *mtrR* promoter −35A Δ and the 23S rRNA C2611T mutations, which contribute to their AZIR (Fig. 5).

Clade D (*n* = 34) is much more diverse, with various NG-MASTs identified in all 4 years of the study but most prevalent in 2018 (52.9%, 18/34). Over half (52.9%, 18/34) were identified in western Canada, with the remaining in central Canada. Isolates from males made up 61.8% (21/34), with 19.0% (4/21) rectal isolates. The *mtrR* promoter −35A Δ or 23S rRNA C2611T mutations caused AZIR in this clade. One cluster of concern within clade D consists of NG-MAST 5308 isolates (*n* = 6) that were identified in 2018 in central and western Canada. They were AZIR and had decreased susceptibility to ESCs. Their AZIR was due to three 23S rRNA alleles with the C2611T mutation. Five out of the 6 isolates (83.3%) were from females, and four (66.7%) were from pharyngeal infections (Fig. 5).

## DISCUSSION

In Canada, the increase of AZIR N. gonorrhoeae between 2015 and 2018 is primarily due to the multidrug-resistant NG-MAST 12302 clone and STs that are in its genogroup, totaling 2,250 or 65.2% (2,250/3,447) of all study isolates. The mosaic structure of the MtrCDE efflux pump is the mechanism of the low-level AZIR in these isolates (24, 25). This is in contrast to previous years when the 23S rRNA C2611T mutation was the primary source of AZIR (20).

Over 80% of ST-12302 isolates were from males, with approximately 20% identified from rectal infections suggesting a gay, bisexual, and other population of men who have sex with men. These proportions are similar for all of our study isolates with the mosaic *mtr* locus. Isolates without the mosaic locus had slightly lower proportions (approximately 65% males with 16% rectal infections). The development of AZIR in rectal infections is possibly due to the high bioavailability of AZI in rectal tissues (29, 31).

In 2015, ST-12302 was only identified in central Canada, but it spread to western and eastern regions in 2016. The proportion of ST-12302 female infections increased from 7.9% (3/38) in 2015 to 14.1% (47/333) in 2018, indicating its expansion into the heterosexual population.

The pervasiveness of this clone may be due to increased biological fitness caused by the same mechanism that causes AZIR. The overexpression of the mutated MtrCDE efflux pump causes enhanced survival of N. gonorrhoeae in the presence of human neutrophils (32). In contrast, the 23S rRNA mutations appear sporadically and may have a fitness cost (26).

Of interest, ST-12302 is a result of 2 existing alleles (*porB*-908 and *tbpB*-267) combining as opposed to a newly mutated *porB* or *tbpB* allele. ST-12302 has the same *porB* as the internationally identified clone ST-1407 known for high-level resistance to ESCs (33), which was prevalent in Canada from 2010 to 2012 (34). ST-1407 isolates were primarily susceptible to AZI and did not have the mosaic *mtr* locus. While ST-1407 prevalence has decreased since 2012, *porB*-908 persisted and was identified in other STs such as ST-10451 (MDR and the third most prevalent ST in Canada from 2014 to 2015) and now ST-12302. The *tbpB*-267 allele was first seen in Canada in 2012 in 2 isolates from central Canada that were AZI susceptible. It was not identified in Canada again until 2015 in the first ST-12302 isolate, which was AZIR and had the mosaic *mtr* locus. There has been no published data regarding *tbpB*-267 identification in other countries.

While ST-12302g is still the predominant genogroup identified in our study isolates, it has decreased from 80.1% (941/1,175) in 2017 to 54.0% (803/1,486, *P* < 0.05) in 2018, coinciding with the emergence and increase of ST-14994 (2.1%, 24/1,137 in 2017 to 19.8%, 291/1,466 in 2018) and ST-16288 (0.5%, 6/1,137 in 2017 to 5.9%, 87/1,466 in 2018). These STs are not part of ST-12302g but do have the *lactamica*-like mosaic *mtrR* promoter and *mtrD* alleles (Pathogenwatch’s mtrD mosaic 2; mtrR promoter mosaic 2).

ST-14994 differs from ST-12302g in that it has the A39T *mtrR* mutation instead of the mosaic D79N/S183N/M197I, has a partial-mosaic *mtrE* allele, and has a PorB1a protein structure (Table 3). Isolates of this group tend to have a lower average AZI MIC, most likely due to the absence of the mosaic *mtrR* mutations (24) and possibly due to the lack of *porB* mutations that have an additive effect on AZIR (21). The PorB1a protein structure increases the isolate’s resistance to complement-dependent killing in normal human serum (35) and is often identified in disseminated gonococcal infections (DGIs). Five DGIs were associated with ST-14994 study isolates in 2018 and are cause for concern, as DGIs are on the rise in Canada. In 2015, 0.3% (4/4,190) of isolates submitted to the NML were DGIs; in 2019, it increased to 1.4% (70/4,859) (National Microbiology Laboratory; I. Martin, P. Sawatzky, and G. Liu, unpublished data). The United States has also identified DGIs in 2019 and 2020 with the same PorB1a structure and MLST as Canadian ST-14994, though NG-MAST was not cited (36).

ST-16288, identified in 2017 and 2018, also has a slightly lower average AZI MIC (Table 3) than ST12302g isolates, which may be due to the absence of a *porb1b* G120 mutation that is present in ST-12302g isolates. ST-16288 isolates were primarily identified in western Canada (92.5%, 86/93), with only 7.5% (7/93) found in central Canada. There is no published data that NG-MAST 16288 has been identified outside Canada.

AZIR N. gonorrhoeae associated with mosaic *mtr* alleles has been documented in the United States (26, 27), Australia (28), and Germany (29). Germany also identified NG-MAST 12302g as the predominant clone with the mosaic *mtr* locus from 2016 to 2018 (29). Other countries that have ST-12302 isolates listed in Pathogenwatch include Ireland (2015), the United Kingdom (2016), the United States (2016), Australia (2016), and Norway (2017) (37), signifying the spread of this lineage globally. The ST-14994 clone has also been identified in the United States (2016), Norway (2017), and Australia (2017) (37), but these did not have the *mtrR* promoter and *mtrD* mosaics that were identified in Canadian ST-14994 isolates with AZI MICs of ≥1 mg/L. Canadian ST-14994 isolates with an AZI MIC of <0.5 mg/L (not included in this study) also did not exhibit mosaic *mtr* alleles, suggesting these mutations were acquired after this clone emerged in Canada. The first Canadian ST-14994 N. gonorrhoeae, identified in May 2017, had AZI MICs of 0.125 to 0.25 mg/L. In September 2017, ST-14994 isolates with AZI MICs of 1 mg/L and mosaic *mtrR* promoter and *mtrD* alleles emerged. While the more resistant version of ST-14994 is more prevalent, the other strain is still identified in Canada.

High-level AZIR is also a concern both internationally (38–45) and here in Canada (1). The 5 high-level AZIR isolates identified in Canada during our study period were isolated from 2016 to 2018 in western and central regions. Four were isolated from the pharynx, where AZI is less effective and commensal *Neisseria* transfer of resistance determinants and reduced tissue penetration of AZI can lead to increased resistance (46). High-level AZIR is primarily due to the 23S rRNA A2059G mutation; however, a new mutation, A2058G, which confers similar resistance, was identified in the United States between 2016 and 2019 (22). This mutation was not identified in our study isolates.

In the last 20 years, we have seen a decline in the proportion of cultures received at the NML due to the shift to nucleic acid amplification testing (NAAT) for the diagnosis of gonorrhea in lieu of culturing. This is especially an issue for the smaller provinces and the remote regions of Canada, as culturing and shipping N. gonorrhoeae is difficult. Provinces/territories that culture N. gonorrhoeae follow regional protocols for doing so that are often dictated by risk factors of the patient and site of infection. Most of the larger centers that do their own AST primarily send cultures that are nonsusceptible to the NML for testing. As cultures of N. gonorrhoeae are required for AST and WGS, all regions of Canada, as well as populations that are not high risk for sexually transmitted infections, may not be represented equally. This problem can also cause the proportions of AZIR to be overrepresented in some regions (for example, the eastern region) or not identified in some regions (such as the northern region).

Another limitation of this study is our lack of resources to perform WGS on all cultures received at the NML, and while we endeavored to choose a representative number of isolates from the different regions and with various NG-MASTs, we focused on resistant isolates and, therefore, could not compare them to a representative number of susceptible isolates. Also, we looked for specific mutations that have been identified to affect AZI susceptibility and may have missed mutations that have not been previously documented.

The clonal dissemination of ST-12302 occurred across Canada and was also identified internationally. It is possible that this clone emerged due to sublethal azithromycin concentrations triggering an N. gonorrhoeae isolate which already has the widespread *porB*-908 allele to mutate to an ST-12302 clone in the presence of the commensal Neisseria lactamica. While ST-12302 has the *lactamica*-like mosaic *mtr* locus, a *meningitidis*-like mosaic *mtr* locus has also been identified as a contributor to AZIR (25), although not in Canada. The increased biological fitness of this clone and others with the mosaic *mtr* locus enhances their ability to survive and spread through sexual networks. Monitoring of antimicrobial susceptibilities and sequence types of N. gonorrhoeae and using WGS to provide molecular antibiotic resistance, virulence, and fitness determinants can help us understand the higher transmission rates of certain lineages and track their spread. Combining phenotypic and molecular testing will help us monitor emerging mutations and their effects on AMR and transmission. Identification of specific mutations associated with antimicrobial resistance is necessary to support the development of NAAT assays for AMR prediction and point-of-care tests. Data generated from this surveillance are used to update the CGSTI with the most effective treatment given current antimicrobial resistance and, consequently, reduce the spread of drug-resistant gonorrhea. These guidelines are currently under review to address the increase in AZIR in Canada.

## MATERIALS AND METHODS

### Antimicrobial susceptibility testing and molecular testing.

Between 2015 and 2018, 19,625 N. gonorrhoeae isolates were tested nationally, either in provincial laboratories or at the National Microbiology Laboratory (NML). The NML has antimicrobial susceptibility data for 14,816 of these isolates, NG-MAST sequence types for 11,993 isolates, and WGS data for 2,763 isolates (Table 1).

MICs were determined using agar dilution following CLSI methodology (47) or Etest (48). Isolates with an AZI MIC of ≥1 mg/L were included in this study (*n* = 3,447). Molecular genotyping using the NG-MAST method was successfully performed on 98.0% (3,377/3,447) of them as previously described (49). ST genogroups were defined as STs that differed by less than 5 bp. WGS was performed as previously described (20) on 1,544 of these isolates (2015, *n* = 56; 2016, *n* = 105; 2017, *n* = 682; 2018, *n* = 701), selected to include a convenience sample of geographical and temporal distribution of isolates as well as a range of NG-MAST and MLST sequence types (STs) (Table 1). A subset of these isolates can be found in BioProject under accession no. PRJNA785548. ST-12302 isolates that were identical according to the WGS data analyzed (mutations and molecular typing) were represented by one isolate in this subset to reduce the number of isolates uploaded to 715.

Study isolates were grouped into regions based on the province they were identified in: the western region included isolates from British Columbia, Alberta, Saskatchewan, and Manitoba; central region isolates were from Ontario and Quebec; and eastern region isolates were from Nova Scotia and New Brunswick.

### Whole-genome sequencing and assembly.

The WGS analyses were conducted on 1,544 AZIR N. gonorrhoeae isolates collected from provinces in Canada at the NML as previously described. DNA samples were prepared using Epicentre MasterPure Complete DNA and RNA extraction kit (Mandel Scientific, Guelph, Ontario, Canada), and libraries were created with Nextera sample preparation kits (Illumina, San Diego, CA) with 300-bp paired-end indexed reads generated on the Illumina NextSeq platform (Illumina, San Diego, CA). The quality of the reads was assessed using FastQC version 0.11.4 (https://www.bioinformatics.babraham.ac.uk/projects/fastqc/) and assembled using Shovill (Galaxy Version 1.0.4+galaxy) (50). Core single nucleotide variant (SNV) phylogenetic analysis was conducted using a custom Galaxy SNVphyl (50) phylogenomics workflow (Galaxy version SNVPhyl v1.0.1b Paired-end) using NCCP11945 (GenBank accession no. NC_011035) as a mapping reference with thresholds of minimum coverage of 7, minimum mean mapping quality of 30, and alternative allele ratio of 0.75 and removing highly recombinant regions containing >5 SNVs per 500 bp. Phylogenetic trees were visualized using FigTree v1.4.3 (http://tree.bio.ed.ac.uk/software/figtree/). Phylogenetic clades were determined visually and correlated by cluster analysis using ClusterPicker (51) with the following settings: initial and main support thresholds, 0.9; genetic distance threshold, 0.045; and the large cluster threshold, 10.

The WGS data were used to determine MLST and NG-STAR ST designations as well as the detection of molecular antimicrobial resistance markers (17–25), including the mosaic *mtrR* promoter; mosaic *mtrR*, *-C*, -*D*, -*E*, and -*F* alleles; the *mtrR* −35A Δ; MtrR A39T and G45D mutations in the MacAB efflux pump; 23S rRNA A2058G, A2059G, and C2611T mutations (E. coli numbering A2058G, A2059G, and C2611T are A2044G, A2045G, and C2567T, respectively, in N. gonorrhoeae NCCP11945); mutations in *rplD* (L4) and *rplV* (L22); and the presence of a Correia element or *erm* (A, B, C, F), *ere* (A and B), *mefA*, and *mphA* genes (20).

Pathogenwatch (pathogen.watch) (30) was used to produce a core distance-based neighbor-joining tree of 911 Canadian NG-MAST 12302 isolates with WGS data. Pathogenwatch was also used to confirm AMR determinant genes and mutations identified (https://cgps.gitbook.io/pathogenwatch). Clades were determined visually and confirmed with the ClusterPicker program (51) using a main support threshold of 0.9, a genetic distance threshold of 4.5, and large cluster threshold of 10.

### Statistical analysis.

Absolute and relative frequencies were calculated for categorical variables. Fisher’s exact test was applied to proportion comparisons of AZIR between years with a 95% confidence interval using EpiCalc 2000 (version 1.02; Brixton Health).

### Data availability.

A subset of the isolates used in this study was deposited in BioProject under accession no. PRJNA785548.
